# Longitudinal dyadic associations between psychological stress and sadness rumination in young and middle-aged stroke patients and their spouses: an actor-partner interdependence model study

**DOI:** 10.3389/fpubh.2026.1910517

**Published:** 2026-09-11

**Authors:** Yue Wu, Chang Liu, Yi-hao Wu, Dao-fei Ji, Yong-mou Sun, Ru-fei Dai

**Affiliations:** 1 Department of Neurosurgery, The Second Affiliated Hospital of Xuzhou Medical University, Xuzhou, Jiangsu, China; 2 Department of Neurosurgery, Xuzhou Central Hospital, Xuzhou, Jiangsu, China

**Keywords:** actor-partner interdependence model, cross-lagged analysis, longitudinal studies, psychological stress, sadness rumination, stroke

## Abstract

**Aim:**

To explore the longitudinal associations between psychological stress and sadness rumination in young and middle-aged stroke patients and their spouses based on the actor-partner interdependence model.

**Methods:**

The convenience sampling method was used to select 256 stroke patients and their spouses as the participants. The Stanford Acute Stress Reaction Questionnaire and the Rumination on Sadness Scale were used to investigate the patients at the acute stage (T1), 3 months (T2), and 6 months (T3) after stroke. Equivalence testing and cross-lag analysis of the actor–partner interdependence model were used for statistical analysis.

**Result:**

256 dyads (512 individuals) were enrolled; 230 dyads (460 individuals, 89.8% retention) completed all three waves after excluding 26 dyads due to stroke recurrence (*n* = 5), death (*n* = 2), and loss to follow-up (*n* = 19). The results of the equivalence test showed that the psychological stress and sadness rumination of patients and their spouses met the Configural Invariance, Metric Invariance, and Scalar Invariance across time (∆CFI ≤ 0.01). Actor effects: patient stress→subsequent rumination (*β* = 0.24, 95% CI [0.18, 0.30], *p* < 0.001; *β* = 0.20, 95% CI [0.14, 0.26], *p* < 0.001); spouse stress→subsequent rumination (*β* = 0.21, 95% CI [0.15, 0.27], *p* < 0.001; *β* = 0.18, 95% CI [0.12, 0.24], *p* < 0.001). Partner effects: spouse rumination→patient rumination (*β* = 0.17, 95% CI [0.10, 0.24], *p* < 0.01; *β* = 0.15, 95% CI [0.08, 0.22], *p* < 0.01).

**Conclusion:**

Young and middle-aged stroke patients and their spouses exhibited high temporal stability in psychological stress and sadness rumination, with significant bidirectional actor effects and asymmetric partner effects. Longitudinal associations indicated a significant positive spillover effect, wherein spouses’ sadness rumination prospectively predicted patients’ sadness rumination.

**Limitations:**

Single-center recruitment, unmeasured confounders (rehabilitation intensity, medication adherence), no gender-stratified partner-effect test.

## Introduction

Stroke in young and middle-aged patients has a rapid onset, a long course of disease, and a high disability rate, often causing severe shocks to the patient and their family system (1, 2). Different from elderly patients, young and middle-aged patients are in the peak period of their careers and bear the heavy burden of family responsibilities. Compared with older stroke survivors (>60 years) who have already retired and assumed a diminished caregiving role at home, middle-aged and younger stroke survivors are typically at the peak of their careers and bear substantial family responsibilities. As primary breadwinners of their households, they often shoulder dual duties of raising children and supporting aging parents. The sudden onset of functional impairment signifies not only a loss of physical capacity but also a disruption of social roles and a destabilization of their status as economic pillars (3, 4). Consequently, disease-induced career interruption and social role loss can trigger distinct stress responses (5). In this extreme stress situation, the psychological stress responses of patients and their spouses, as a closely coupled dyadic whole, do not exist in isolation but are intertwined and dynamically evolving (6, 7). Previous studies mostly focused on the post-traumatic stress symptoms or depression of individual patients (8, 9), ignoring the hidden psychological burden borne by the spouse as the primary caregiver and lacking in-depth discussions on the dynamic association mechanism between the two during the disease adaptation process (10). Although cross-lagged network analysis has revealed the dynamic association characteristics of post-stroke depressive symptoms (11), few studies have employed a binary framework to capture the bidirectional influence between patients and their spouses—this study aims to fill this gap. Therefore, from the perspective of the dyadic relationship, it is of great significance to clarify the evolution of the psychological stress of patients and their spouses over time and its internal action path for the implementation of precise family intervention.

The Actor–Partner interdependence model breaks through the traditional individualistic research paradigm and emphasizes that in intimate relationships, the outcome variables of individuals are affected by both their own predictors and their partner’s predictors (12). In this framework, the patient’s level of psychological stress is not only determined by their own sadness rumination but may also be affected by the spillover effect of the spouse’s psychological state (13). As a non-specific state of physical and mental tension, psychological stress is manifested as fear of disease uncertainty, anxiety about losing control of future life, and denial of self-worth in this specific population of young and middle-aged stroke patients (14). And sadness rumination, namely repeatedly engaging in passive thinking about a traumatic event, is seen as a key cognitive mechanism for maintaining and increasing psychological stress (15). Unlike adaptive rumination, sadness rumination is often trapped in ineffective cyclic thinking about causes and consequences, hindering the process of emotional repair (16).

This study aimed to construct a longitudinal tracking design to reveal the association trajectory of psychological stress and sadness rumination in patients and their spouses during the critical window from the acute phase to the recovery phase. We hypothesized that at different stages after stroke, the levels of psychological stress of patients and their spouses would show different changes, and there would be a significant positive correlation between them. Due to the greatest impact in the early stage of the illness, the psychological stress of both parties may peak in the early stage of hospitalization and then gradually decline as rehabilitation progresses (17). However, the rate at which the spouse’s stress levels drop could be lagging behind, and there may even be a rebound at a certain stage, which is associated with the cumulative effect of the long-term care load.

Based on this, the following research hypotheses are proposed in this study:

*Hypothesis* 1: The psychological stress and sadness rumination of young and middle-aged stroke patients and their spouses have high temporal stability within 6 months, and there is a significant positive correlation between them.

*Hypothesis* 2: The Actor effect is significant, that is, the psychological stress of patients and their spouses can positively predict their own subsequent sadness rumination. At the same time, the sadness rumination of both parties can also positively predict their own subsequent psychological stress, forming a self-reinforcing vicious circle.

*Hypothesis* 3: The Partner effect is significant, that is, the psychological stress and sadness rumination of patients and their spouses can cross individual boundaries and mutually predict each other’s psychological symptoms. Specifically, the spouse’s psychological stress and sadness rumination will positively predict the patient’s psychological stress and sadness rumination, and the patient’s psychological stress and sadness rumination will positively predict the spouse’s psychological stress and sadness rumination.

In view of this, this study captures the dynamic changes through longitudinal data and uses the Actor–Partner interdependence model to analyze the two-way association between pairwise data to understand the level of psychological adaptation of young and middle-aged stroke patients and their spouses. This not only helps to identify the characteristics of high-risk families and clarify the key targets of intervention but also provides a solid empirical basis for the construction of preventive psychological support programs from the dyad level, and ultimately promotes the positive adaptation and reconstruction of the whole family system.

## Participants and methods

### Participants

Using the convenient sampling method, a total of 256 stroke patients and their spouses admitted to the Department of Neurosurgery of the Second Affiliated Hospital of Xuzhou Medical University from January 2025 to October 2025 were selected as the research subjects.

Inclusion criteria:

Patients met the diagnostic criteria of stroke, were diagnosed as having a stroke (18) by head CT, MRI and other imaging examinations, and had their first attack.

The patient and their spouse were all 18–59 years old.

The patient’s vital signs are stable, and the Montreal Cognitive Assessment (MoCA) score is ≥ 18 points, ensuring that the patient has sufficient cognitive function to understand and complete the questionnaire items.

The patients and their spouses had basic abilities of listening, speaking, reading and writing without severe aphasia or dysarthria. They could complete the questionnaire independently or with a little assistance from the researcher.

Written informed consent was obtained from both patients and their spouses.

Exclusion criteria:

Patients and their spouses with severe heart, liver, kidney and other important organ failure.

Patients with malignant tumor, severe infection or acute autoimmune disease.

Patients and their spouses had a history of psychiatric disorders or were taking antipsychotic drugs.

This study has received ethical approval from the Second Affiliated Hospital of Xuzhou Medical University (approval number: xkzy202510325).

### Calculation of sample size

Equation for repeated measurement sample size (17, 19).


$$n=\frac{2}{\delta^{2}}[\sigma_{\mu }^{2}+\frac{1+(K-1)p_{c}}{K}{\sigma_{e}}^{2}]{\left(U_{\alpha /2}+U_{\beta }\right)}^{2}$$

Where $\rho_{c}$ denotes the intraclass correlation coefficient, derived from a pilot study conducted by our team involving 42 stroke patient-spouse dyads (ICC for psychological stress = 0.52); δ represents the target effect size, set at 0.22 based on analogous dyadic cross-lagged studies; $\sigma_{\mu }^{2}$ indicates inter-individual variance, and ${\sigma_{e}}^{2}$ indicates intra-individual residual variance, both sourced from the pilot study. With α = 0.05 and β = 0.10, the calculation indicated a minimum requirement of 182 dyads. Accounting for an anticipated 10% attrition rate in longitudinal studies, the minimum sample size was set at *n* = 182÷(1–10%) = 203. The present study initially enrolled 256 dyads, with a final effective sample of 230 dyads. Although the sample size is relatively modest, it meets the minimum requirement for statistical testing. Furthermore, the use of Full Information Maximum Likelihood (FIML) estimation effectively preserved statistical power despite missing data.

### Survey tools

#### General information questionnaire

The general information questionnaire was composed of two parts:

1. The general demographic data (age, gender, education level, pre-illness occupational status, monthly income, medical insurance type) and disease-related information (stroke type, NIHSS score, Barthel index) of stroke patients.
2. Spouse demographic data (age, gender, education level, occupational status, monthly income, medical insurance type), health status (number of chronic diseases), and care status (daily care hours, whether the sole caregiver).

#### Stanford acute stress reaction questionnaire

The Chinese version of the SASRQ scale was used in this study (20). The scale comprises five dimensions: dissociative symptoms, re-experiencing of the traumatic event, avoidance behaviors, anxiety/hyperarousal, and social functional impairment, with 30 items scored on a 0–5 scale (total range 0–150). At the acute stroke phase (T1), the scale assessed acute stress reactions. At the sub-acute (T2) and recovery phases (T3), given stroke as a persistent stressor, scores reflected sustained psychological stress levels. Previous studies have validated the sound psychometric properties of this scale across different post-stroke timepoints. The scale has been validated in Chinese populations with trauma and stroke, demonstrating a Cronbach’s α of 0.930 and a test–retest reliability coefficient of 0.980.

#### Rumination on sadness

Developed by Abela et al., this study selected the sadness rumination subscale related to sadness-based reflection for relevant adjustment and measurement (21). It includes 13 items. Each item is scored from 0 to 3, ranging from “almost never” to “almost always,” and the total score ranges from 0 to 39. The Rumination Response Scale (RRS) was sinicized following a standard forward–backward translation procedure and further adapted cross-culturally by our research team. It demonstrated sound psychometric properties in Chinese adults with chronic diseases, with a Content Validity Index (CVI) of 0.92 and a Cronbach’s α of 0.786, meeting established measurement standards.

Both stroke patients and their spousal caregivers completed identical versions of the Stanford Acute Stress Reaction Questionnaire (SASRQ) and the RRS. Apart from modifications in the instructions to clearly specify the referent (i.e., patients reporting on their own condition and spouses reporting on their own condition), the item wording remained consistent across both groups. Although systematic psychometric evidence specific to dyadic stroke samples remains limited, these instruments have previously been applied in studies involving Chinese patients with neurological disorders and their family members, demonstrating strong ecological applicability. Furthermore, the results of measurement invariance testing conducted in this study—establishing configural, metric, and scalar invariance—provide empirical support for the comparability of scores between patients and spouses, thereby justifying cross-subject score interpretation.

### Data collection methods

Using convenience sampling, we consecutively enrolled stroke patients and their spouses meeting the inclusion criteria from the Department of Neurosurgery, Second Affiliated Hospital of Xuzhou Medical University, between January and October 2025. Following approval by the Hospital Ethics Committee, all participants provided written informed consent. Prior to data collection, the principal investigator provided systematic training to all research assistants to standardize protocols, unify questioning phrasing, and prohibit leading questions, ensuring homogeneity and rigor in data acquisition. General demographic data and clinical baseline information (disease duration, lesion location, NIHSS score, Barthel Index, etc.) were extracted from the electronic medical record system to minimize recall bias.

The timing of psychological stress and sadness rumination assessments was determined based on the phased trajectory of post-stroke psychological adaptation: (1) T1 (Acute phase): 7–14 days post-stroke, once the patient’s vital signs were stable, coinciding with the peak of acute stress reactions and psychological distress (22); (2) T2 (Sub-acute phase): 3 months (12–14 weeks) post-stroke, representing a high-risk period for negative emotions such as depression and anxiety, overlapping with the critical window for functional rehabilitation; (3) T3 (Recovery phase): 6 months (24–26 weeks) post-stroke, when most mild-to-moderate psychological stress begins to remit alongside functional recovery, serving as a key node for evaluating long-term adaptation trajectories (23). Surveys at each timepoint were conducted in a private, quiet environment. Data were anonymized, and participants were explicitly informed that their responses would be used solely for scientific research purposes.

### Statistical methods

Before data entry, the original data were independently checked by two people to ensure accuracy, and then the database was established. The full information maximum likelihood (FIML) estimator in Mplus 8.3 was used to handle missing data. FIML yields unbiased parameter estimates under the assumption of missing at random (MAR). Compared with listwise deletion, FIML better preserves statistical power and reduces estimation bias. GraghPad 10.0 software was used for descriptive statistical analysis. The count data were expressed as frequency (percentage) [%]. After a normal distribution test, the measurement data were expressed as the mean ± standard deviation, and for non-normal distribution, the median (quartile) was used. Mplus 8.3 software was used to test the measurement equivalence of longitudinal data. A morphological equivalence model, a factor loading equivalence model, and an intercept equivalence model were established to verify that the measurement of the patient and his/her spouse on the same latent variable had cross-time and cross-Actor invariance.

On this basis, the Cross-lagged APIM model was constructed to investigate the longitudinal associations of psychological stress and sadness rumination between patients and their spouses at T1, T2, and T3, respectively. In the model, both psychological stress and sadness rumination were modeled using Latent Variables to improve the estimation accuracy. The Actor Effect was defined as the influence of an individual’s own psychological stress on their own sadness rumination at a subsequent time point. The Partner Effect was defined as the influence of the individual’s psychological stress on the spouse’s sadness rumination at a subsequent time point.

## Results

### General information of the respondents

During follow-up, 26 patients were lost to follow-up due to recurrent stroke (*n* = 5), unexpected death (*n* = 2), refusal to answer phone calls (*n* = 10), and refusal to respond to survey items (*n* = 9). Comparisons of demographic and clinical characteristics between the dropout group (*n* = 26) and the retained group (*n* = 230) revealed no significant differences in age, sex, NIHSS scores, Barthel Index, or baseline psychological measures (all *p* > 0.05), suggesting no substantial attrition bias. Missing data were handled using full information maximum likelihood (FIML) in Mplus 8.3. Little’s MCAR test indicated χ^2^ = 128.34, df = 112, *p* = 0.12, supporting the assumption of data being missing at random (MAR). Details in Table 1.

**Table 1:** General information of young and middle-aged patients and their spouses (*n* = 230).

Patient information	Classification	n	Percentage (%)	Patient information	Classification	n	Percentage (%)
Age (years)	< 45	67	29.13	Occupational status	On-the-job	105	45.65
	45–59	163	70.87		Leaving a job	73	31.74
Gender	Male	136	59.13		Retirement	52	22.61
	Female	94	40.87	Type of insurance	Resident insurance	113	49.13
Education level	Junior high school and below	123	53.48		Employee health insurance	117	50.87
	High school	69	30.00	NIHSS score	1–4	53	23.04
	College or above	38	16.52		5–15	89	38.70
Monthly income (yuan)	< 2000	17	7.39		16–20	71	30.87
	2000 -	89	38.70		≥21	17	7.39
	5,000 -	92	40.00	Barthel index	61–99	93	40.43
	> 8,000	32	13.91		41–60	124	53.91
Types of stroke	Bleeding	158	68.70		≤40	13	5.65
	Ischemic	72	31.30				
Spouse information	Classification	n	Percentage (%)	Spouse information	Classification	n	Percentage (%)
Age (years)	< 45	68	29.57	Monthly income (yuan)	< 2000	16	6.96
	45–59	162	70.43		2000 -	84	36.52
Gender	Male	94	40.87		5,000 -	99	43.04
	Female	136	59.13		> 8,000	31	13.48
Education level	Junior high school and below	120	52.17	Number of chronic diseases	< 3	156	67.83
	High school	70	30.43		≥3	74	32.17
	Junior college and above	40	17.39	Hours of care (h/d)	> 12	89	38.70
Occupational status	On-the-job	110	47.83		6–12	95	41.30
	Leaving	65	28.26		< 6	46	20.00
	Retirement	55	23.91	Sole caregiver	yes	116	50.43
Type of health care	Resident insurance	110	47.83		no	114	49.57
	Employee health insurance	120	52.17				

### Common method bias test

In this study, all variables were collected using a self-administered scale. To test whether there was a serious Common Method Bias (CMB), according to Podsakoff et al. (24), an unrotated principal component factor analysis was performed on the data of all three longitudinal measurements via the Harman single-factor test. The results showed that the variance explanation rates of the first factor extracted from the three measurements were 14.67% (T1), 18.94% (T2), and 23.50% (T3), respectively, all of which were far below the critical standard of 40% (25). This indicates that there is no significant common method bias in this study, and the data quality is reliable.

### Scores and correlation analysis of psychological stress and sadness rumination in young and middle-aged stroke patients and their spouses

The scores of each scale are presented in Table 2, and the correlation was significant (*p* < 0.05).

**Table 2:** Psychological stress and sadness rumination scores of young and middle-aged stroke patients and their spouses and their correlation analysis (*r* value, *n* = 230).

Items	M, SD	①	②	③	④	⑤	⑥	⑦	⑧	⑨	⑩	⑪	⑫
①Patients’ psychological stress T1	124.35 ± 10.36	1											
②Patients’ psychological stress T2	112.24 ± 11.88	0.585^**^	1										
③Patients’ psychological stress T3	100.06 ± 10.09	0.515^**^	0.532^**^	1									
④Spouses’ psychological stress T1	121.22 ± 10.08	0.500^**^	0.461^**^	0.314^*^	1								
⑤Spouses’ psychological stress T2	110.63 ± 12.04	0.433^**^	0.502^**^	0.383^**^	0.528^**^	1							
⑥Spouses’ psychological stress T3	96.35 ± 9.25	0.412^**^	0.455^**^	0.444^**^	0.495^**^	0.408^**^	1						
⑦Patients’ sadness rumination T1	30.05 ± 3.57	0.562^**^	0.463^**^	0.357^*^	0.312^*^	0.315^*^	0.298^*^	1					
⑧Patients’ sadness rumination T2	26.34 ± 3.70	0.502^**^	0.446^**^	0.362^**^	0.359^**^	0.384^**^	0.344^*^	0.523^**^	1				
⑨Patients’ sadness rumination T3	21.48 ± 3.24	0.401^**^	0.407^**^	0.439^**^	0.406^**^	0.312^**^	0.443^*^	0.434^**^	0.502^**^	1			
⑩Spouses’ sadness rumination T1	28.24 ± 3.69	0.499^**^	0.412^**^	0.324^*^	0.344^*^	0.405^**^	0.286^*^	0.468^**^	0.476^**^	0.324^*^	1		
⑪Spouses’ sadness rumination T2	24.19 ± 3.58	0.432^**^	0.451^**^	0.316^**^	0.362^**^	0.429^**^	0.312^*^	0.412^**^	0.483^**^	0.303^*^	0.402^*^	1	
⑫Spouses’ sadness rumination T3	18.93 ± 3.20	0.368^**^	0.413^**^	0.436^**^	0.406^**^	0.435^**^	0.402^*^	0.365^**^	0.375^**^	0.364^**^	0.367^*^	0.406^*^	1

^*^p < 0.05, ^**^p < 0.01.

### Equivalence test measurement

In this study, multiple-group confirmatory factor analysis was used to test the measurement equivalence of the psychological stress scale and the sadness rumination scale of patients and their spouses across time. Morphological equivalence, factor loading equivalence, and intercept equivalence models were constructed in sequence, and the change of the fit index of the nested models was compared (see Table 3). The results showed that, compared with the baseline model, the ΔCFI of each restricted model was ≤0.01, and the changes of RMSEA and SRMR were all within the acceptable range, indicating that the psychological stress and sadness rumination scales of patients and their spouses had cross-time measurement equivalence at the three time points. The results show that the same construct has the same structural connotation, factor meaning, and the same zero-point reference standard at different time points and for different actors, which meets the metrological premise of the cross-lagged Actor–Partner interdependence model analysis.

**Table 3:** Equivalence model fitting results of three measurements of psychological stress and sadness rumination of patients and their spouses.

Items	Classification	*χ^2^*	*df*	*p*	CFI	∆CFI	TLI	RMSEA	SRMR
Patients’ psychological stress	Configural Invariance	212.140	72	< 0.001	0.986	-	0.980	0.036	0.022
	Metric Invariance	248.269	76	< 0.001	0.985	0.000	0.982	0.034	0.023
	Scalar Invariance	296.357	84	< 0.001	0.981	0.004	0.980	0.040	0.024
Spouses’ psychological stress	Configural Invariance	204.125	72	< 0.001	0.985	-	0.981	0.037	0.031
	Metric Invariance	236.733	76	< 0.001	0.982	0.000	0.980	0.038	0.032
	Scalar Invariance	278.802	84	< 0.001	0.979	0.002	0.979	0.038	0.034
Patients’ sadness rumination	Configural Invariance	305.543	102	< 0.001	0.977	-	0.965	0.046	0.035
	Metric Invariance	340.804	110	< 0.001	0.973	0.000	0.971	0.046	0.037
	Scalar Invariance	385.456	122	< 0.001	0.972	0.003	0.965	0.047	0.036
Spouses’ sadness rumination	Configural Invariance	300.289	102	< 0.001	0.976	-	0.962	0.055	0.054
	Metric Invariance	332.422	110	< 0.001	0.971	0.002	0.964	0.053	0.055
	Scalar Invariance	373.538	122	< 0.001	0.971	0.006	0.961	0.054	0.055

χ^2^ is chi square, df is degrees of freedom, CFI is comparative fit index, ∆CFI is variation comparative fit index, TLI is non-benchmarking fit index, RMSEA is root mean square of progressive residual, SRMR is standardized root mean square residual.

### Cross-lag analysis of the actor-partner interdependence model of psychological stress and sadness rumination in young and middle-aged stroke patients and their spouses

Establishment and fitting of psychological stress and sadness rumination models for young and middle-aged stroke patients and their spouses.

In this study, first, an unconstrained cross-lagged Actor-Partner interdependence Model (Model M1) was constructed. In the model, the error of the same observed variable at three measurement points was allowed to be correlated, and the latent variables (psychological stress and sadness rumination) at the same time point were allowed to be correlated.

In order to further test the homogeneity and symmetry of the pairwise data, a limiting Model (Model M2) was constructed based on M1. That is, the parameters of the model were restricted to cross-Actor equivalence:

The autoregressive effects and Actor effects of the patients and their spouses on the same variables were restricted to be equal;

The Partner effects of patients and their spouses on the same variables were limited to be equal;

The equality of Actor and Partner effects between different variables was restricted.

The comparison results of model fitting are shown in Table 4. Compared with M1, although the Δχ^2^ (Δdf) of M2 reached the significance level, its ΔCFI was ≤0.01, and RMSEA and SRMR had little change, indicating that the fitting difference between M2 and M1 was within the acceptable range.

**Table 4:** Fitting results after limiting the actor-partner interdependence model of psychological stress and sadness rumination for young and middle-aged stroke patients and their spouses.

Model	*χ^2^*	*df*	*p*	CFI	∆CFI	TLI	RMSEA	SRMR
M1: Saturation model of psychological stress and sadness rumination in young and middle-aged stroke patients and their spouses	1679.373	680	< 0.001	0.966	-	0.956	0.040	0.066
M2: Bounded model	1866.284	702	< 0.001	0.964	0.001	0.955	0.036	0.058

χ^2^ is chi square, df is degrees of freedom, CFI is comparative fit index, ∆CFI is variation comparative fit index, TLI is non-benchmarking fit index, RMSEA is root mean square of progressive residual, SRMR is standardized root mean square residual.

Based on the principle of conciseness, the more restrictive interpretation of M2 did not significantly reduce the model and could more clearly reveal the common mechanism of paired variables. So, M2 was selected as the final model for this study.

Path analysis of cross-lagged Actor–Partner interdependence model of psychological stress and sadness rumination in young and middle-aged stroke patients and their spouses.

Measurement invariance across time was tested separately for stroke patients and their spouses on the Stanford Acute Stress Reaction Questionnaire (SASRQ) and the Rumination–Reflection Scale (RRS) using Mplus 8.3. A series of nested models were sequentially estimated: configural invariance (identical factor structure across time), metric invariance (equal factor loadings, indicating consistent factor meaning), and scalar invariance (equal intercepts, ensuring consistent zero reference points). Invariance was supported if the change in the comparative fit index (ΔCFI) was ≤ 0.01. Results indicated that all four latent constructs met the criteria for scalar invariance (patient psychological stress: ΔCFI = 0.004; spouse psychological stress: ΔCFI = 0.002; patient rumination: ΔCFI = 0.003; spouse rumination: ΔCFI = 0.006). These findings support the comparability of latent variable scores across time and across dyad members, satisfying the prerequisite for Actor–Partner Interdependence Model (APIM) analyses.

APIM Specification:

Both psychological stress and rumination were modeled as latent variables: psychological stress was indicated by the 30 items of the SASRQ, and rumination by the 13 items of the RRS. The factor loading of the first item for each latent variable was fixed at 1.0 for scale identification.

The model incorporated three types of pathways: (a) autoregressive paths (e.g., T1 patient psychological stress → T2 patient psychological stress); (b) actor effects (e.g., T1 patient psychological stress → T2 patient rumination); and (c) partner effects (e.g., T1 spouse rumination → T2 patient rumination).

Residual covariances were permitted among latent variables measured at the same time point (e.g., the residual of T1 patient psychological stress with the residual of T1 patient rumination). Residual covariances for the same observed indicators across waves were allowed to correlate only when modification indices provided statistically significant justification, preventing over-parameterization.

Model constraints were implemented as follows: an unconstrained saturated model (M1) was first specified, followed by a constrained model (M2) where: (1) autoregressive effects and actor effects for identical variables were constrained to be equal between patients and spouses; and (2) partner effects for identical variables were constrained to be equal between both dyad members. The plausibility of the constraints was evaluated based on changes in CFI, RMSEA, and SRMR. The more parsimonious M2 was selected as the final model.

**Figure 1:**
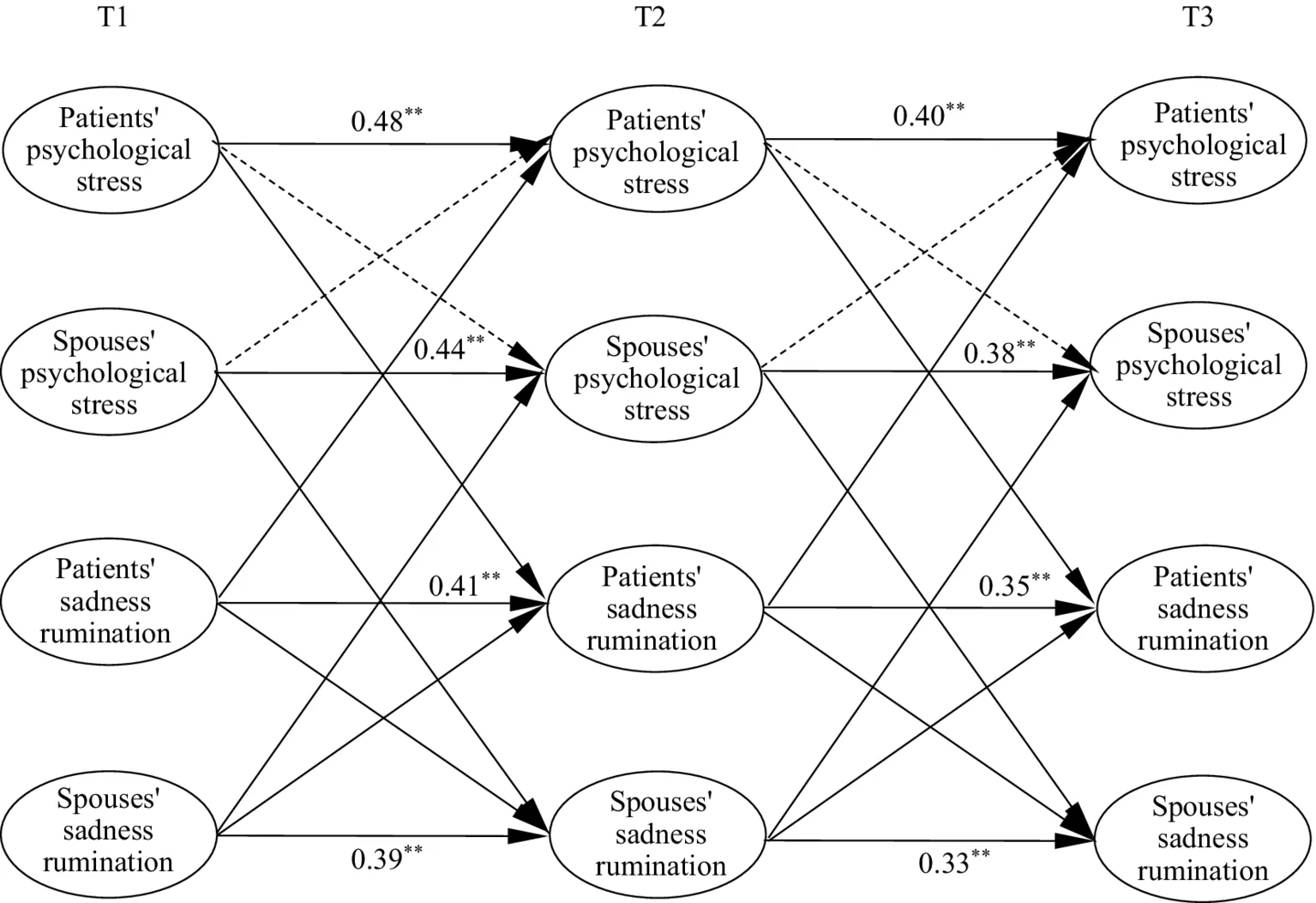
Cross-lagged actor-partner interdependence model of psychological stress and sadness rumination in young and middle-aged stroke patients and their spouses. ^*^p < 0.01, *^*^p < 0.001. Only the path coefficients with statistical significance are marked in the figure, and the dashed line is the path without statistical significance.

The standardized path coefficients of the bounded model M2 are shown in Figure 1. The model results showed that the psychological stress and sadness rumination of young and middle-aged stroke patients and their spouses exhibited strong temporal stability. The autoregression coefficients of each variable ranged from 0.33 to 0.48 (*p* < 0.001), indicating that the mental state at the previous time point could significantly predict the subsequent level.

The psychological stress of young and middle-aged stroke patients and their spouses has a significant Actor effect on sadness rumination. That is, the psychological stress of patients can positively predict the patients’ sadness rumination in the next stage (T1 → T2: *β* = 0.24, T2 → T3: *β* = 0.20, both *p* < 0.001), and the spouse’s psychological stress could positively predict the spouse’s sadness rumination in the next stage (T1 → T2: *β* = 0.21, T2 → T3: *β* = 0.18, both *p* < 0.001).

The effect of patient and spouse sadness rumination on the main body of psychological stress is significant. Specifically, patients’ sadness rumination can positively predict the patient’s psychological stress in the next stage (T1 → T2: *β* = 0.21, T2 → T3: *β* = 0.19, *p* < 0.001), and the spouse’s sadness rumination can positively predict the spouse’s psychological stress in the next stage (T1 → T2: *β* = 0.19, *p < 0.*001; T2 → T3: *β* = 0.16, *p* < 0.01).

The Partner effect of sadness rumination was partially significant. That is, the patient’s sadness rumination could positively predict the spouse’s sadness rumination in the next stage (T1 → T2: *β* = 0.16, *T2 → T*3: *β* = 0.14, both *p* < 0.01), and the spouse’s sadness rumination could positively predict the patient’s sadness rumination in the next stage (T1 → T2: *β* = 0.17, T2 → T3: *β* = 0.16, *p* < 0.01). Actor effects ranged from *β* = 0.16 to 0.24 (95% CI, 0.08–0.31).

Although statistically significant, these represent small-to-medium effect sizes, suggesting that while self-reinforcing cycles exist between individuals’ psychological stress and rumination, other unmeasured factors (e.g., social support, rehabilitation progress) likely play substantial roles. Partner effects ranged from *β* = 0.14 to 0.17 (95% CI, 0.05–0.26), reflecting even smaller effect sizes. This indicates that emotional spillover between spouses is relatively modest. In clinical practice, there is no need to overemphasize the negative impact of spousal emotions; rather, efforts should focus on early identification of high-risk couples (e.g., those with a history of psychiatric disorders in spouses or extremely heavy caregiving burdens). Notably, partner effects for psychological stress were non-significant (*β* = 0.05–0.06, 95% CI: −0.03 to 0.14, *p* > 0.05). This specificity clarifies the interpersonal transmission mechanism: significant dyadic contagion exists solely for rumination, not for overall psychological stress. The effects of Actor and Partner are shown in Table 5.

**Table 5:** Path analysis of cross-lagged actor-partner interdependence model of psychological stress and sadness rumination in young and middle-aged stroke patients and their spouses.

Actor-partner effect	Path	β(T1 → T2)(95% CI)	β(T2 → T3)(95% CI)
Actor effect	Patients’ psychological stress → Patients’ sadness rumination	0.24 (0.12–0.36)**	0.20 (0.09–0.31)**
	Spouses’ psychological stress → Spouses’ sadness rumination	0.21 (0.10–0.32)**	0.18 (0.07–0.29)**
	Patients’ sadness rumination → Patients’ psychological stress	0.21 (0.10–0.32)**	0.19 (0.08–0.30)**
	Spouses’ sadness rumination → Spouses’ psychological stress	0.19 (0.08–0.30)**	0.16 (0.05–0.27)*
Partner effect	Patients’ sadness rumination → Spouses’ sadness rumination	0.16 (0.05–0.27)*	0.14 (0.03–0.25)*
	Spouses’ sadness rumination → Patients’ sadness rumination	0.17 (0.06–0.28)*	0.15 (0.04–0.26)*
	Patients’ psychological stress → Spouses’ psychological stress	0.06 (−0.03–0.15)	0.06 (−0.04–0.16)
	Spouses’ Psychological stress → Patients’ psychological stress	0.05 (−0.04–0.14)	0.05 (−0.05–0.15)

*p < 0.01, **p < 0.001.

## Discussion

This study delineates the longitudinal associations—rather than causal relationships—between psychological stress and rumination within stroke patient-spouse dyads. While the actor effects suggest that higher baseline stress predicts increased subsequent rumination in both partners, these within-person links do not constitute evidence that stress causes rumination. Likewise, the prospective association between spouse rumination and patient rumination does not imply that targeting spousal rumination would necessarily disrupt the dyadic emotional cycle—claims regarding causality or intervention efficacy require validation through randomized controlled trials. Therefore, our findings offer a potential empirical basis for developing future dyadic interventions but fall short of providing definitive evidence for clinical causal effects.

### The development trend of psychological stress and sadness rumination in young and middle-aged stroke patients and their spouses

The results of this study show that the levels of psychological stress and sadness rumination of young and middle-aged stroke patients and their spouses generally show a downward trend during the 6 months after stroke. However, both show strong time stability, indicating the continuous impact of sudden cerebrovascular events on the family system.

In the acute phase (T1), patients face neurological deficit and life threat, while spouses face role transformation and care load. The psychological stress and rumination of both spouses reach the peak at this time (26). With the progress of treatment, although physiological functions gradually recover, the psychological aspects of traumatic memories (such as dissociative symptoms and repeated re-experience) do not disappear immediately upon discharge but transform into continuous sadness rumination (27). This is consistent with the research of Zong Xiaojia et al. on the trajectory of PTSD after stroke in young and middle-aged people (23), suggesting that psychological adaptation is a long-term process. Without intervention, the high mental load in the acute phase can easily evolve into a chronic psychopathological state.

### The actor effect of psychological stress and sadness rumination in young and middle-aged stroke patients and their spouses

This study confirmed that there is a significant two-way Actor effect between psychological stress and sadness rumination. On the one hand, psychological stress significantly and positively predicts subsequent sadness rumination. The stress interaction model theory points out that when an individual’s perceived stress outweighs their coping resources, they will produce a sense of helplessness and then fall into rumination about negative emotions (28). On the other hand, sadness rumination also significantly and positively predicts subsequent psychological stress, indicating that excessive rumination consumes individual cognitive resources, hinders problem-solving, and leads to an increase in an individual’s susceptibility to subsequent stressful events (29). This effect exists independently in patients and their spouses, indicating that the psychopharmacological mechanism is independent at the couple level. Simply improving the psychological state of one partner is not sufficient to break the vicious cycle of the other partner (30).

### The partner effect of psychological stress and sadness rumination in young and middle-aged stroke patients and their spouses

This study found that the Partner effect presents asymmetry. The spouse’s sadness rumination can significantly and positively predict the patient’s sadness rumination (*β* = 0.17–0.15), and the patient’s sadness rumination can also significantly predict the spouse’s sadness rumination (*β* = 0.16–0.14). This indicates that there is a significant Spillover Effect of emotion between spouses. The possible reason for this asymmetry lies in the caregiver centrality in the family system: the emotional state of the spouse, as the main support provider, often determines the emotional climate of the family (31–33). When a spouse is engaged in sadness rumination, the support provided is often negative, fatigued, or even hostile, and this negative association directly exacerbates the psychological distress of the patient (34). In contrast, although the patient is the carrier of the disease, the impact of the fluctuation of their psychological state on their spouse may be more reflected through functional dependence. In terms of emotional transmission, the spouse’s mental health has a more direct and strong projection effect on the patient (35, 36).

### Clinical implications and implementation considerations

Our findings highlight the potential value of integrating dyadic psychological assessment into stroke rehabilitation pathways. However, this should be framed as a future direction rather than an immediate clinical mandate. Given the modest magnitude of partner effects, clinical attention might reasonably prioritize high-risk dyads—such as spouses with a prior history of depression or exceptionally high caregiving burden—rather than universal intensive intervention.

From an implementation perspective, dyadic support could be embedded into existing workflows to minimize resource burden. A pragmatic, tiered approach could involve:

(1) Brief screening with abbreviated stress and rumination scales during acute admission to identify at-risk couples;
(2) Integrating 10–15 min of structured couple communication guidance into routine post-discharge outpatient reviews (e.g., at 1 and 3 months), avoiding the need for standalone intervention units;
(3) Leveraging low-cost digital tools (e.g., WeChat mini-programs delivering emotion regulation skills and peer caregiving experiences) to reduce professional workload.

Such strategies may be particularly feasible in resource-limited primary care settings, where piloting with high-risk dyads first could facilitate the accumulation of localized implementation experience. Nevertheless, the actual effectiveness of dyadic interventions requires confirmation through randomized controlled trials; this study provides a foundation for designing those future evaluations.

### Limitations of the study

This study still has the following limitations. First, the study was only conducted at a single center, and the sample mainly consisted of young and middle-aged patients. This may lead to selection bias and limit the generalization of the conclusions to elderly stroke patients or individuals with other cultural backgrounds.

Secondly, although this study conducted a 6-month longitudinal follow-up, it is still insufficient to understand the long-term evolution of psychological stress and rumination (such as more than 1 year).

Third, we utilized the SASRQ to assess psychological stress at 3 and 6 months post-stroke. While the SASRQ was primarily designed to measure psychological stress reactions in the immediate post-trauma period, its item content (e.g., re-experiencing, avoidance, hyperarousal) stably captures persistent post-traumatic stress symptom clusters in chronic stress research. Future studies should consider combining the SASRQ with instruments such as the PTSD Checklist for DSM-5 (PCL-5) at sub-acute and recovery phases to more precisely track the evolution of symptom profiles.

Future work could employ ecologically valid methods such as daily diaries or extend follow-up periods to better capture the micro-processes governing these dyadic associations. Incorporating physiological markers (e.g., cortisol levels) or neuroimaging metrics would also deepen our understanding of actor–partner interdependence from a biopsychosocial perspective. Furthermore, randomized controlled trials are needed to test whether low-intensity, digitally supported dyadic interventions can be sustainably integrated into routine stroke care without imposing undue burden on clinical workflows.

## Conclusion

Young and middle-aged stroke patients and their spouses exhibited high temporal stability in psychological stress and sadness rumination, with significant bidirectional actor effects and bidirectional partner effects. Longitudinal associations revealed a positive spillover effect of spouses’ sadness rumination on patients’ rumination, with numerically stronger effects from spouses to patients, although this difference was not statistically significant. Clinical practice should establish a couple-centered intervention framework that synchronously addresses the mental health of spouses to disrupt negative dyadic cycles.

## Data Availability

The original contributions presented in the study are included in the article/supplementary material, further inquiries can be directed to the corresponding authors.
